# Cross-Limb Interference during Motor Learning

**DOI:** 10.1371/journal.pone.0081038

**Published:** 2013-12-03

**Authors:** Benedikt Lauber, Jesper Lundbye-Jensen, Martin Keller, Albert Gollhofer, Wolfgang Taube, Christian Leukel

**Affiliations:** 1 Department of Sport and Sport Science, University of Freiburg, Freiburg, Germany; 2 Department of Neuroscience and Pharmacology, University of Copenhagen, Copenhagen, Denmark; 3 Department of Nutrition, Exercise and Sports, University of Copenhagen, Copenhagen, Denmark; 4 Department of Medicine, Movement and Sports Science, University of Fribourg, Fribourg, Switzerland; University G. d'Annunzio, Italy

## Abstract

It is well known that following skill learning, improvements in motor performance may transfer to the untrained contralateral limb. It is also well known that retention of a newly learned task A can be degraded when learning a competing task B that takes place directly after learning A. Here we investigate if this interference effect can also be observed in the limb contralateral to the trained one. Therefore, five different groups practiced a ballistic finger flexion task followed by an interfering visuomotor accuracy task with the same limb. Performance in the ballistic task was tested before the training, after the training and in an immediate retention test after the practice of the interference task for both the trained and the untrained hand. After training, subjects showed not only significant learning and interference effects for the trained limb but also for the contralateral untrained limb. Importantly, the interference effect in the untrained limb was dependent on the level of skill acquisition in the interfering motor task. These behavioural results of the untrained limb were accompanied by training specific changes in corticospinal excitability, which increased for the hemisphere ipsilateral to the trained hand following ballistic training and decreased during accuracy training of the ipsilateral hand. The results demonstrate that contralateral interference effects may occur, and that interference depends on the level of skill acquisition in the interfering motor task. This finding might be particularly relevant for rehabilitation.

## Introduction

It has long been known that the practice of motor tasks with one limb improves not only the performance of the trained but also of the contralateral untrained limb. This has been demonstrated for a number of motor skills ranging from mirror tracing to the exertion of force [1]–[4]. Particularly the practice of ballistic finger movements caused not only an increase in performance (i.e. the rate of force development) of the trained limb but also of the contralateral untrained limb [1]. Previous studies tried to explain the mechanism(s) being responsible for this so called cross-limb transfer (also termed cross-education). Generally, the different hypotheses can be divided into two broad categories: the first category is the bilateral access hypothesis suggesting that adaptations occur in neural networks which are involved in the control of the trained ipsilateral limb but are also accessible to the untrained limb. The second so called cross-activation hypothesis claims that unilateral practice of a motor task causes bilateral motor adaptations [5]. However, the exact mechanisms underlying cross-limb transfer remain elusive and it appears that the amount of cross-limb transfer depends on the nature of the task [6]–[8].

After acquiring a motor task, there are several factors like interference that may degrade the ability to retain the corresponding motor memory. The interference effect refers to the phenomenon of a degradation in the retention of a task A when a different task B is learned after the previously practiced task A [9], [10]. For ballistic motor practice (task A), this interference effect was shown when a second accuracy visuomotor task (task B) was trained directly afterwards [11]. This interference seems to be specific for neural circuits, which are involved in a particular movement and muscle activation [11].

So far, no study investigated possible cross-limb interference effects. Interference effects and their neural correlates [12] have only been investigated for the limb actively involved in the training and it remains therefore to be elucidated whether interference also affects motor performance of the contralateral untrained limb (referred to as cross-limb interference in the following).

The aim of the present study therefore was to test if cross-limb interference effects can be observed in the limb contralateral to the limb that initially practiced a ballistic force task followed by a visuomotor accuracy task. In addition to the evaluation of behavioural changes we also assessed changes in corticospinal excitability using suprathreshold single-pulse transcranial magnetic stimulation (TMS) of the primary motor cortex of the ‘untrained hemisphere’ while subjects practiced the ballistic and the visuomotor interference task.

## Methods

### Subjects

A total number of 55 subjects volunteered to participate in this study. All subjects who participated in this study were right handed according to the Oldfield handedness inventory [13] and gave their written informed consent prior to participation in the experiment. All experiments were generally approved by the ethics committee of the University of Freiburg (54/10) and were in accordance with the Declaration of Helsinki. None of the subjects had any known neurological or orthopaedic disorders.

**Figure 1:**
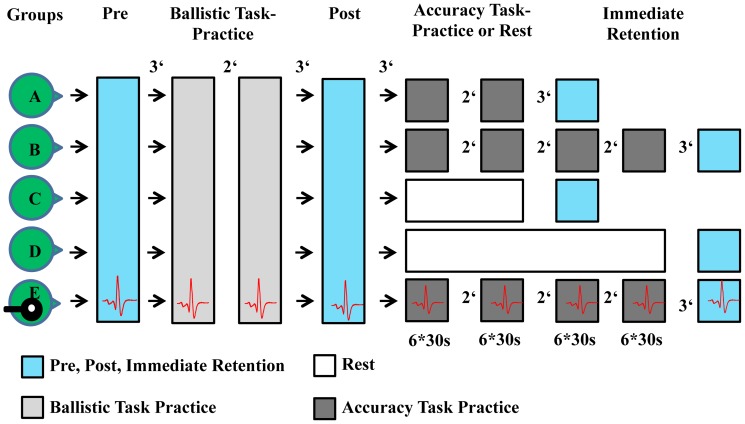
Overview of the study design. All groups (A, B, C, D, E) performed five isometric contractions using their right followed by their left hand index finger. This was followed by a training period of 30 contractions solely executed with the right hand index finger. In the post-test, subjects again performed five contractions with the right hand index finger followed by 5 contractions with their left hand index finger. In the course of the AT, group A practiced the AT with their right hand index finger for 6 minutes and group B for 12 minutes whereas group C rested for 6 minutes and group D for 12 minutes, respectively. Following this, all subjects again performed five contractions with the right and left index finger. Group E executed exactly the same protocol as group B but in addition received transcranial magnetic stimulation.

The 55 subjects who participated in this experiment were randomly allocated to 5 groups consisting of an equal number of 11 subjects (Figure 1). All subjects performed a ballistic movement training (ballistic task, BT). After this ballistic movement training (task A), a period followed where the groups did either practice an interfering accuracy task (AT) with different durations (task B) or rested before again testing the performance in the previously learned ballistic task (task A).

Group A performed a 6 minute AT (**Group A**; 24.1±2.6 years, 7 women), group B a 12 minute AT (**Group B**; 25.5±2.6 years, 4 women).

Group C rested for 6 minutes (**Group C**; 26.2±4.4, 5 women), and group D rested for 12 minutes (**Group D**; 25.5±2.5; 4 women)

In the 5^th^ group E, we applied single-pulse TMS after each executed trial to evaluate changes in corticospinal excitability in the ipsilateral (untrained) hemisphere with respect to the trained limb during and following ballistic motor training and training of the AT. Group E executed the AT for 12 minutes (**Group E**; 24.7±2.5 years, 4 women).

### General experimental procedure

The ballistic task and the AT were always accomplished first by isometric flexions of the right index finger followed by flexions of the left index finger using a custom built robot. The subjects were seated in an adjustable chair with their forearms fixed (right or left) in a custom built arm and hand rest to prevent wrist and arm movements. The subject's index finger was fixed to a splint mounted on the arm of the robot. The axis of rotation of the robot arm was aligned with the metacarpophangeal joint of the subject's right or left hand so that the centre of rotation of the robot arm corresponded to the joint centre of the subject's finger. The applied force when flexing the index finger was recorded by a torquemeter (LCB 130, ME-Meβsysteme, Neuendorf, Germany) mounted in the robot arm. Before the actual experiment started, subjects were instructed to perform 10 submaximal contractions (index finger flexions, arm pronated) in a self-paced frequency as warm up with each hand separately. After this warm up, the actual experiment began and the subjects always started to perform the BT.

### Ballistic task (BT)

BT consisted of isometric ballistic contractions aiming to improve the rate of force development of the contractions. This task was chosen as it was previously shown that similar tasks caused rapid improvements in motor performance [1], [5]. Before the testing session started, subjects were instructed how to perform the task and were allowed two test contractions with maximal intensity. Subjects were instructed to produce as much lateral force as fast as possible solely by flexing their index finger. These contractions occurred in response to auditory cues: At the beginning of each contraction, subjects heard a tone (100 ms, 500 Hz sine) signalling them to get ready followed by a second tone 2 seconds later (200 ms, 600 Hz sine wave) being the start signal for the ballistic isometric contraction. Subjects were instructed to initiate the contraction immediately with the second tone (within 250 ms). Thus, subjects were instructed to wait for the second tone but after a couple of trials, they were able to anticipate the second tone and to perform a contraction that was as long as the tone (200 ms).

It was previously shown that providing augmented feedback about the maximum velocity of the contraction (rate of force development, RFD) proved to be effective to improve the performance e.g. [1], [11]. Therefore, subjects received post-trial visual feedback on their RFD calculated from the force-time curve and presented as a number on a computer screen placed 1 m in front of the subjects. Feedback was provided 1 second after subjects finished their contraction and lasted for 4 seconds. Subjects were instructed to perform their maximum in every trial and to increase their RFD (i.e. try to increase the number shown on the computer screen) on every subsequent trial throughout the training. Subjects were also verbally encouraged throughout the training.

The contractions were performed every 5 seconds. Initially, subjects performed 5 contractions without augmented feedback that served as baseline value with the right hand followed by 5 contractions with the left hand index finger. Thereafter, subjects executed a total of 30 contractions with augmented feedback (training) including a rest of 2 minutes after the initial 15 contractions exclusively with their right dominant hand. After the training and a rest of 3 minutes, subjects performed another 5 contractions with the right hand followed by 5 contractions with the left hand again without visual feedback. No feedback was given in the post test as we wanted to exclude the influence of augmented feedback on performance as changes in performance under the influence of feedback do not always reflect learning [14]. The 5 contractions without visual feedback for the right and the left hand were repeated after the subjects in the different groups performed a subsequent AT (see below) or rested for a defined time (6 or 12 minutes), which was equivalent to the time they trained the AT.

### Accuracy task (AT)

The isometric AT involved visuomotor tracking of a computer generated sinusoid curve and was carried out exclusively with the right hand. The sinusoid curve comprised alternating sine waves of different frequencies ranging from 0.5 to 3 Hz and the duration of the constructed sinusoid curve was 30 seconds. There were two periods of null potentials with a duration of 2 seconds (one occurring in the middle of the 30 seconds sequence and the second at the end of the sequence). The subjects were instructed to relax their muscle and rest during the period with null potential. The sinusoid curve was presented on the same computer screen as used for the BT. The curve was a running black line from the right to the left side with a visible sequence of 6 seconds. At the trough of the sine wave with the lowest amplitude, a red line indicated the force output of the subjects when flexing their index finger.

Subjects were instructed to keep this red line as close as possible to the black target line by isometric contractions with the right hand index finger pushing against the robot arm. Thus, like in the BT, performance in the AT depended on augmented feedback and on the activation of muscles responsible for flexing the right hand index finger. The force that needed to be applied to match the peak of the highest sine was 9 N, meaning that the required force to accomplish the task was very low and that a precise adjustment of motor output was necessary for executing the task. This was in strong contrast to the BT.

The training sequence of 30 seconds was repeated 12 times (training duration of 6 minutes) or 24 times (training duration of 12 minutes). Subjects were verbally encouraged to improve their performance every subsequent trial. After subjects completed the AT 6 times they were allowed to rest for 2 minutes.

Subjects were given a rest of 2 seconds after practicing the AT for 15 seconds and at the end of every AT trial to exclude fatiguing effects and to ensure no EMG activity for the TMS measurements (see *Recordings and Stimulation procedure*). Subjects were instructed to keep the force signal as close as possible to the target line by submaximal (∼10% of initial maximal contractions) pressing the robot arm and were verbally encouraged to improve their performance every subsequent trial.

### Recordings and stimulation procedure

#### Transcranial Magnetic Stimulation

For group E, transcranial magnetic stimuli were applied over the right hemisphere motor cortex using a Magstim Rapid Rate Stimulator (Magstim® Company Ltd., Whitland, UK) with a figure of eight coil (Magstim SP 16097). For each subject, the initial stimulation point was set approximately 0.5 cm anterior to the vertex and over the midline. The final position for the stimulation was determined by moving the coil anterior and right from the vertex while the MEP size of the first dorsal interosseus muscle (FDI) of the untrained hand was monitored (induced current was anterior-posterior, coil arm was tilted 45 degrees below horizontal). Resting motor threshold (MT) was determined as the lowest intensity to evoke MEPs >50 µV in at least three out of five sweeps in the left FDI [15], [16]. The optimal position for eliciting MEPs in the FDI with minimal stimulator intensity was marked with a felt pen directly on the subject's head. The coil position relative to the head was permanently checked throughout the experiment to ensure a constant position of the coil relative to the stimulation site. Stimulation intensity for the experimental protocols (BT and AT) was adjusted to 130% MT (corresponding to ∼50% maximum stimulator output) evoking always clear MEPs in the FDI EMG.

For the BT, TMS was applied 3 seconds after subjects finished the contraction and stimulation was applied after each executed trial (5 pre trials, 30 learning trials, 5 post trials, 5 immediate retention trials).

During the AT, TMS was applied during the two resting periods occurring in the middle and at the end of the sequence of 30 s (see *Accuracy Task*). Stimulation was triggered 1 second after the rest period started. The FDI background EMG was monitored by the experimenter to ensure that the background EMG remained silent around the time of stimulation. In case of muscular activation, the trial was excluded from analysis and the experimenter reminded the subject to relax in the consecutive trials with stimulation. According to the aforementioned procedure, subjects were stimulated 48 times during the AT.

#### EMG

For the TMS protocol in group E, surface EMG was obtained from FDI of the left hand using bipolar surface electrodes (Blue sensor P, Ambu®, Bad Nauheim, Germany). The interelectrode distance was 1.5 cm. The reference electrode was placed on the olecranon of the same arm. The EMG recordings were amplified (x 1000), bandpass filtered (1–10 kHz) and sampled at 2000 Hz. All data was stored on a computer using custom-built software (LabView based, National Instruments, Austin, TX) for off-line analysis.

### Data Analyses and Statistics

#### BT

Motor performance in the BT was determined as the rate of force development (RFD) in a time window around the force produced by the subjects. The RFD was defined as the maximal slope of the force time curve (dT/dt) in each trial [17]. The RFD mean was calculated for the 5 pre trials, 5 post trials and 5 immediate retention trials. Changes in performance from pre to post were assed by comparing the pre with the post values. To test for the effect of the AT on BT performance (i.e. interference), we compared the BT post performance values with the BT immediate retention performance values. The RFD values obtained during the training were normalized to the RFD of the first contraction of the training for each subject. This normalization of motor performance to baseline was performed in order to allow comparison

#### AT

Motor performance (movement error) in the AT was calculated as the mean absolute difference between the force curve produced by the subjects and the target sinusoid curve over periods of 30 seconds (30 seconds indicates one trial, see *Accuracy Task*). All obtained values (12 versus 24 values, see *Accuracy task*) were normalized to the value of the first trial. For quantification of changes in performance, the average of the initial four values during training was compared to the average of the final four values.

#### TMS

MEP size was calculated offline as peak-to-peak amplitude in a time window from 5 ms after the stimulation until the end of the MEP. To quantify the changes in MEPs during training, the average of the initial four MEP amplitudes was compared to the average of the final four MEP amplitudes for BT and AT, respectively.

#### Statistics

Before all statistical comparisons, normal distribution of the data was tested using Shapiro- Wilks test. All statistical comparisons were made using performance data normalized to baseline but results are also presented as percentage change to aid description of the data.

Before normalizing the data, differences in baseline performance (pre-test) was excluded by calculating separate one-way ANOVAs for the trained and untrained hand using non-normalized data.

After normalizing the data to baseline, a three way repeated measures ANOVA was conducted with factors TIME (post, immediate retention), GROUP (A, B, C, D, E) and HAND (trained, untrained).

Changes in in the BT and AT were calculated using separate measures of ANOVA with factors TIME (initial four values, last four values) and GROUP (A, B, C, D, E). Changes due to the BT training were analysed by a two-way ANOVA with factors TIME (initial four values, last four values) and GROUP (A, B, C, D, E) and changes due to the AT with factors TIME (initial four values, last four values) and GROUP (A, B).

In the case of significant interactions, Bonferroni corrected t-tests were calculated to identify changes within the groups.

For group E, correlation between possible changes in performance and changes in corticospinal excitability were computed by Person's correlation tests. All other results obtained for group E were calculated using paired Student's T-test.

All data are presented as percentage change in means + standard error of the mean (SEM).

## Results

Before the training, there were no significant differences between the groups A, B, C and D for the trained (group: F_3,30_ = 0.47; p = 0.69) as well as for the untrained hand (group: F_3,30_ = 0.59; p = 0.62).

### Pre versus Post

#### BT performance

From pre to post, there was a significant increase in ballistic performance during the course of the training when comparing the initial 4 contraction with the last four contractions (TIME F_1,4_ = 129.856 η^2^ = 0.722; p<0.001). This increase was comparable across groups (TIME x GROUP F_1,4_ = 1.023, η^2^ = 0.076; p = 0.405).

### Post versus Immediate Retention

#### BT performance

**Figure 2:**
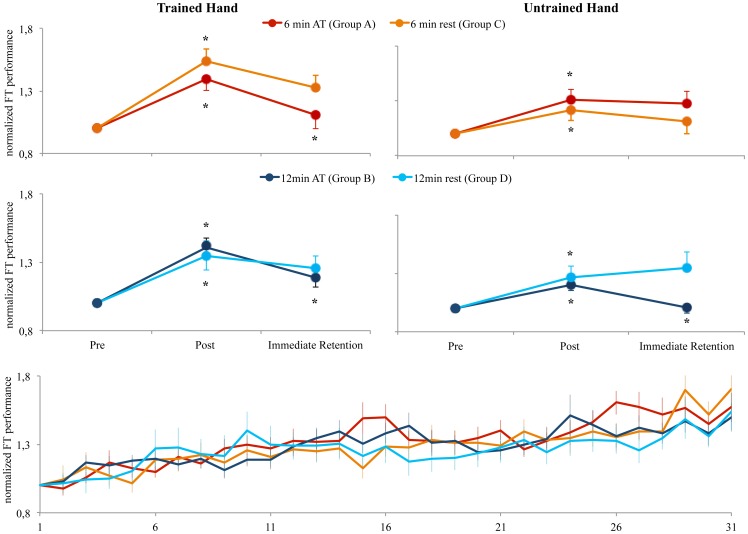
Changes in BT performance in the pre, post and immediate retention test across groups. All groups (A, B, C, D) significantly (indicated by *) increased their performance from the pre- to the post-test in the trained as well as in the untrained hand. After the AT, groups A (AT for 6 minutes) and B (AT fro 12 minutes) showed a significant reduction in BT performance in the immediate retention test of the trained hand. However, only group B showed a significant reduction in BT performance in the immediate retention test in the untrained hand. The box at the bottom shows the increase in BT performance over the training period for groups A, B, C, D.

From post to immediate retention, there was a significant interaction between time and groups (TIME x GROUP F_1,4_ = 3.849, η^2^ = 0.235; p = 0.008) which was also significantly different between hands (TIME x HAND x GROUP F_1,4_ = 3.023, η^2^ = 0.195; p = 0.026). After the completion of the accuracy training, only the groups who actually practiced the accuracy task (Group A 6 minutes of accuracy training, Group B 12 minutes of accuracy training and Group E 12 minutes of accuracy training) showed a significant reduction in ballistic performance in the trained hand (Figure 2; Group A −28.94±3.59, p = 0.027; Group B −22.10±4.17%, p = 0.026, Group E −18.00±3.63%, p = 0.01). The other groups who rested instead of training the accuracy task (C 6 minutes rest, D 12 minutes rests) did not show significant changes in ballistic performance (Group C+20.84±5.87%, p = 0.23; Group D+9.00±3.67%, p = 0.89).

For the untrained hand, however, only groups A and E, which trained the accuracy task for 12 minutes displayed significant reductions in ballistic motor performance in the immediate retention test (Figure 2; Group A −19.13±4.85%, p = 0.007; Group E −22.42±8.17%, p = 0.02). All other groups which either trained the accuracy task for 6 minutes (Group A), rested for 6 minutes (Group C) or rested for 12 minutes (Group D) showed no significant changes in ballistic motor performance (A −3.40±6.04%, p = 0.58; Group C+10.34±5.69%, p = 0.20; Group D −8.10±7.25%, p = 0.30).

#### AT performance

**Figure 3:**
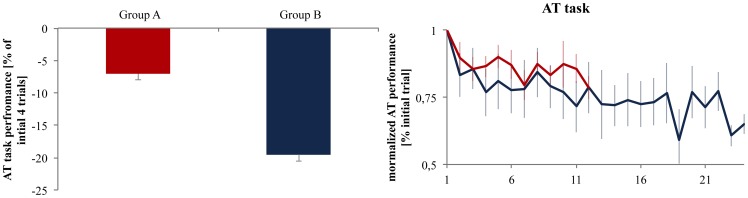
Changes in performance in the AT. Group A and B reduced their movement error in the course of the AT training (expressed as percentage change compared to the initial four trials). However, group B practicing the AT for 12 minutes showed a significant (indicated by *) greater reduction in movement error compared to group A training for only 6 minutes. The right box displays the change in AT over the training period.

**Figure 4:**
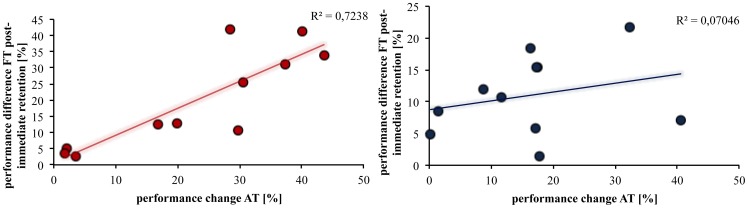
Correlation between BT and AT. Correlation between the performance change in the BT from post to immediate retention and the change in performance in the AT for groups B (left) and A (right). There was a significant correlation between the reduction in movement error in the AT carried out by the trained hand and the decrease in performance of the BT only for group B carried out by the untrained hand.

All three groups (A, B, E) practicing the accuracy task showed a significant increase (TIME F_1,1_ = 18.342, η^2^ = 0.396; p<0.001) in performance when comparing the initial 4 contractions with the last four contractions. To identify if there was an effect of the amount of training on the accuracy task performance, we compared group B training for 12 minutes and group A training only for 6 minutes. This comparison revealed a significant difference between them (TIME x GROUP F_1,1_ = 4.149, η^2^ = 0.172; p = 0.05) as group B showed a greater increase in accuracy task performance (reduction in movement error) than group A (Figure 3; Group A −7.05±3.26%, Group B −19.56±4.76%, p = 0.05). This is further supported by a positive correlation (Figure 4) between the decrease in BT motor performance (difference from post to immediate retention) and the increase in AT motor performance (corresponds to a decrease in in movement error) found for group B (R^2^ = 0.87; p = 0.002) but not for group A (R^2^ = 0.17; p = 0.74).

### Changes in corticospinal excitability associated with BT and AT of the untrained hand

**Figure 5:**
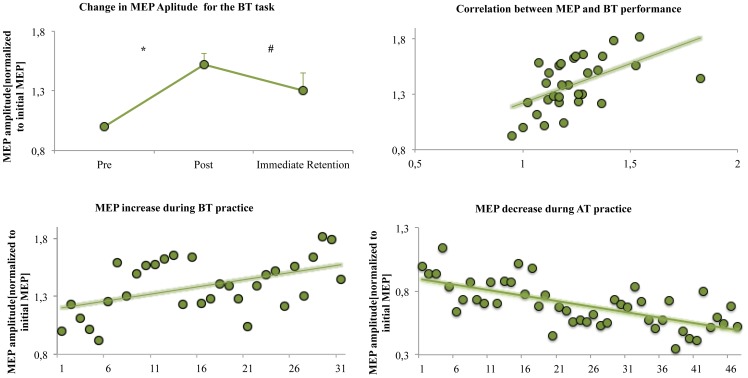
Changes in motor evoked potentials in the pre-post and immediate retentions tests. The size of the MEP significantly increased (indicated by *) in the course of the BT and was still high in the immediate retention test. Furthermore, there was a significant correlation between the increase in the MEP and the increase in BT performance. During the course of the AT, there was a decrease in the MEP.

During ballistic task training of group E, the MEP recorded from the FDI of the untrained hand significantly increase in size in the course of the ballistic training (mean of the last 4 values of the training versus the mean of the first 4 values; +52.07±9.65%, average increase from 1.19±0.11 mV to 1.76±0.08 mV; t_11_ = −7.5; p = 0.002) and were still increased by 30.35±14.44% in the immediate retention test (average 1.51±0.14 mV; t_11_ = −2.44; p = 0.07, Figure 5). Furthermore, there was a correlation between the increase in MEP amplitude and the increase in BT performance of the untrained hand in the course of the BT task (R^2^ = 0.61; p = 0.001, Figure 5).

During the AT training, the corresponding MEPs recorded from the FDI of the untrained hand did significantly decrease in the course of the AT (mean of the last 4 values of the AT training versus the mean of the first 4 values, −41.57±3.59%, t_11_ = 5.46, p = 0.012). There was a trend towards a correlation between the decrease in MEP amplitude and the improvement in AT performance (R^2^ = 0.37; p = 0.07).

## Discussion

In the present study, unilateral motor training was shown not only to improve the performance of the trained but also of the contralateral untrained side. Such a (cross-limb) transfer effect has previously been shown for many tasks from mirror drawing to ballistic contractions [1]–[4]. What was not known so far but demonstrated in the present study is that besides beneficial cross-limb transfer effects, there can also be degrading cross-limb interference effects if additional, competing motor learning training follows initial learning. This cross-limb interference effect, which we document in the present study, is important to consider when conceptualizing motor practice (e.g. training sessions), and it may have wide implications e.g. in neurorehabilitation.

### Cross-limb transfer

Currently, there are two types of hypotheses trying to explain the underlying mechanisms of cross-limb transfer. One of them, the cross-activation hypothesis, states that unilateral practice causes not only an increased motor activity in the contralateral hemisphere controlling this limb but also in the ipsilateral hemisphere [1], [5], [18], [19]. In the present study, the TMS results obtained during the training of the BT task are in line with this cross-activation hypothesis as they demonstrate an increase in corticospinal excitability in the neural circuits being involved in the control of the opposite untrained limb. In this respect, the current study supports the assumption that cross-activation relates to tasks that require a high level of force production, i.e. a strong descending drive [1], [4], [5], [20]–[24]

### Cross-limb interference

The current study shows that unilateral practice of different motor tasks can lead to both cross-limb transfer and cross-limb interference effects. Interestingly, the cross-limb interference effect depends on the level of skill acquisition in the newly acquired (interfering) task. Subjects in the present study trained a visuomotor accuracy task (AT) either for 6 or 12 minutes. The group which trained for 12 minutes did not only obtain a significant better performance, only in this group there was a positive correlation between the increase in AT performance and the subsequent decrease in BT performance (i.e. interference) measured in the retention test. The reason for claiming that it was the level of skill acquisition and not simply the passage of time which caused forgetting of the BT is that there was no significant decrease in performance between the post and the immediate retention tests in the groups who did not practice the AT but rested for 6 and 12 minutes, respectively.

One possible explanation for the cross-limb interference effect might be that the motor memory of task A is fragile in the early phase following learning indicating that consolidation has not ended [9], [11], [25] meaning that the first memory is going to be consolidated while the second memory is being encoded. It was speculated that the process of encoding of the second memory interacts and disrupts the consolidation of the first memory resulting in compromised recall of the first memory [26]. So far, this was only demonstrated for the limb which was actively involved in practicing the competing tasks but not for the contralateral one as indicated by the present results. It might therefore be that the same mechanism causes the contralateral interference effect presented in this study.

### The role of the corticospinal pathway during cross-limb interference

Not only M1 of the ipsilateral [23], [27]–[29] but also of the contralateral side [1] was argued to be involved in ballistic motor learning tasks and this is why the corticospinal excitability on the untrained side was monitored in the present study. We hypothesized that the MEPs in the untrained FDI would increase over the time course of the BT as such an increase was already shown in a previous study [1]. The present results indeed confirm that the corticospinal excitability increases during BT practice. Furthermore, our results demonstrate a correlation between the gain of the MEP amplitude and performance improvements. In addition, the corticospinal excitability was reduced in the time course of the AT, and the results revealed a trend to significance when correlating the corticospinal excitability and the improvements in AT performance. However, this time, the performance gains were negatively correlated with the size of the MEP. This observation is in line with previous studies reporting reduced corticospinal excitability after exercising with low contraction strengths [30]–[32]. Therefore, it seems as if both training adaptations are accompanied by task specific (short-term) adaptations of electrophysiological variables, i.e. the MEP. Alternatively one may speculate that the sequence of tasks determined the direction of adaptation, i.e. the first task increased the MEPs while the second task decreased the MEP size. As we have not measured the tasks in the opposite order we cannot reject this possibility. Nevertheless, based on the above-cited literature concerning intensity specific modulation of corticospinal excitability, this latter explanation seems less likely.

Importantly, as both the initially learned BT and the subsequent newly learned AT are skill tasks and both tasks were carried out in the same movement direction by the same muscles, it is likely that overlapping neural circuitries were involved in the execution of those tasks. Previously it was demonstrated that consolidation, a process where a motor memory is stabilized and becomes less susceptible to interference [33], [34], can be disrupted when the second interfering task activates the same neural circuits [11]. The reciprocally modulated ipsilateral corticospinal excitability, i.e. the increase over time in the BT and a decrease in the AT, might reflect such a differential activation of one and the same structure (M1 selectively and/or its corticospinal connections).

It has to be emphasized that the strong correlation between the increase in AT performance for the trained limb and the decrease in ballistic motor performance for the untrained limb was only seen in the group that trained the AT for 12 minutes but not for the group that only trained for 6 minutes. This implies that in order for interference to occur, there has to be competing learning to a certain extent. Thus, cross-limb interference is not just a matter of performing a subsequent new or unfamiliar task [11]. This was demonstrated by the group that practiced the AT for 12 minutes and showed higher performance values (i.e. a greater reduction in movement error) than the group training the AT only for 6 minutes. For the former group, the increase in AT performance correlated with the decrease in BT performance in the retention test, suggesting that the level of the acquisition in the AT determines the interference effect. Again, this goes well together with the idea that the cross-limb interference relies – at least in part – on usage of the same neural structures in the two tasks. Thus, the longer practice and the consequential greater learning effects of task B (AT) implicates a longer involvement of these overlapping neural circuits likely casing plastic changes in these areas. The result of this longer involvement is a more pronounced interference with task A (BT).

## Conclusion

The results of the present study show that subsequent learning of two unimanual motor tasks can be accompanied not only by cross-limb transfer of the learning effects to the contralateral, untrained limb, it may also be accompanied by cross-limb interference after learning the second task. This cross-limb interference effect depends on the amount of skill acquisition in the interference task. The finding of cross-limb interference following motor skill learning can have important consequences for the strategy in rehabilitation training (e.g. in hemiparesis after stroke). It is consequently important to consider the risk of cross-limb interference when training of the healthy limb is applied in order to influence rehabilitation of the affected limb.
