# Correction: Cross-Limb Interference during Motor Learning

**DOI:** 10.1371/journal.pone.0095813

**Published:** 2014-04-21

**Authors:** The PLOS ONE Staff

The labels for the y-axes are incorrect for Figure 2 and Figure 4. "FT" should be replaced with "BT." The R^2^ values for Figure 4 are also incorrect. The correct R^2^ values are provided in the Results section.

**Figure 2:**
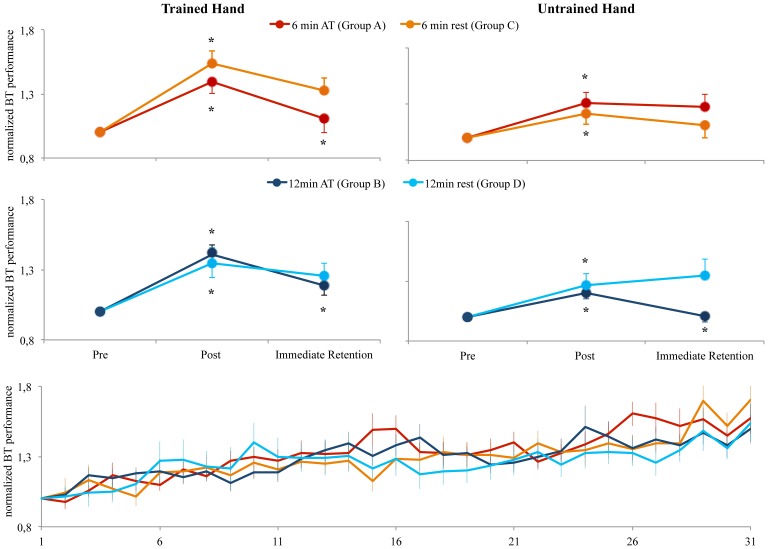
Changes in BT performance in the pre, post and immediate retention test across groups. All groups (A, B, C, D) significantly (indicated by *) increased their performance from the pre- to the post-test in the trained as well as in the untrained hand. After the AT, groups A (AT for 6 minutes) and B (AT for 12 minutes) showed a significant reduction in BT performance in the immediate retention test of the trained hand. However, only group B showed a significant reduction in BT performance in the immediate retention test in the untrained hand. The box at the bottom shows the increase in BT performance over the training period for groups A, B, C, D.

**Figure 4:**
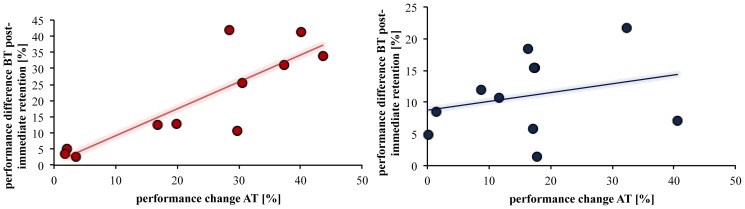
Correlation between BT and AT. Correlation between the performance change in the BT from post to immediate retention and the change in performance in the AT for groups B (left) and A (right). There was a significant correlation between the reduction in movement error in the AT carried out by the trained hand and the decrease in performance of the BT only for group B carried out by the untrained hand.

Please see the corrected Figure 2 here.

Please see the corrected Figure 4 here.
